# Preoperative thrombin generation is predictive for the risk of blood loss after cardiac surgery: a research article

**DOI:** 10.1186/1749-8090-8-154

**Published:** 2013-06-12

**Authors:** Yvonne Bosch, Raed Al Dieri, Hugo ten Cate, Patty Nelemans, Saartje Bloemen, Coenraad Hemker, Patrick Weerwind, Jos Maessen, Baheramsjah Mochtar

**Affiliations:** 1 Department of Cardiothoracic Surgery, Cardiovascular Research Institute Maastricht, Maastricht University Medical Center, P. Debeyelaan 25, PO Box 5800, 6202, AZ, Maastricht, the Netherlands; 2 Synapse BV, Maastricht University Medical Center, PO Box 616, 6200, MD, Maastricht, the Netherlands; 3 Department of Internal Medicine, Maastricht University Medical Center, P. Debeyelaan 25, PO Box 5800, 6202, AZ, Maastricht, the Netherlands; 4 Department of Epidemiology, Maastricht University Medical Center, PO Box 616, 6200, MD, Maastricht, the Netherlands

**Keywords:** Bleeding, Blood (anti)coagulation, Blood loss, Thrombin generation, CABG, CPB

## Abstract

**Background:**

In this study the value of thrombin generation parameters measured by the Calibrated Automated Thrombography for prediction of blood loss after cardiac surgery with cardiopulmonary bypass was investigated.

**Methods:**

Thirty male patients undergoing first-time coronary artery bypass grafting were enrolled. Blood samples were taken pre-bypass before heparinisation (T1) and 5 min after protamine administration (T2). Thrombin generation was measured both in platelet-rich plasma and in platelet-poor plasma. Besides thrombin generation measurements, activated clotting time, haematocrit, haemoglobin, platelet number, fibrinogen, antithrombin, D-dimers, prothrombin time and activated partial thromboplastin time were determined. Blood loss was measured and the amount of transfusion products was recorded postoperatively until 20 hours after surgery. Patients were divided into two groups based on the median volume of postoperative blood loss (group 1: patients with median blood loss <930 ml; group 2: patients with median blood loss ≥930 ml).

**Results:**

On T1, patients of group 2 had a significantly lower endogenous thrombin potential and peak thrombin (p<0.001 and p=0.004 respectively) in platelet-rich plasma, a significantly lower endogenous thrombin potential (p=0.004) and peak thrombin (p=0.014) in platelet-poor plasma, and a lower platelet count (p=0.002). On T2 both endogenous thrombin potential and peak thrombin remain significantly lower (p=0.011 and p=0.010) in group 2, measured in platelet-rich plasma but not in platelet-poor plasma. In addition, platelet number remains lower in group 2 after protamine administration (p=0.002).

**Conclusions:**

The key finding is that the Calibrated Automated Thrombography assay, performed preoperatively, provides information predictive for blood loss after cardiac surgery.

## Background

Blood loss and thrombotic incidents still belong to the most frequent and feared complications in cardiac surgery with cardiopulmonary bypass (CPB) [1], despite of preoperative evaluation of the haemostatic condition by assessment of medical history and common coagulation tests [2,3]. Disturbed haemostasis can be attributed to abnormal thrombin generation (TG), platelet dysfunction and excessive fibrinolysis. Anticoagulation with high doses of heparin may result in further haemostasis impairment. Currently, tools to adequately predict haemostasis and therefore to control blood loss are lacking. Although it was already shown that the use of thromboelastography (TEG) may help to reduce blood product consumption, studies investigating the relationship between coagulation tests, like TEG, haemostatic variables and bleeding are inconsistent in demonstrating a clear predictive value for the volume of blood loss after CPB [2,4,5]. In part, this inconsistency may be due to the fact that TEG only shows clot formation, not the total amount of thrombin, which plays a central role in haemostasis [6].

In this study, we aimed at investigating the value of thrombin generation parameters as measured by the Calibrated Automated Thrombography (CAT) [6] for the prediction of blood loss after cardiac surgery. This assay displays not only the moment blood starts to clot but also the amount of thrombin that forms in clotting plasma with or without platelets. Conceptually, results of CAT measurements correlate with impaired haemostasis if too low, and with thrombosis risk if too high [7,8]. CAT might improve the timely identification of haemostatic problems underlying a bleeding tendency perioperatively. In this study, we hypothesize that CAT measurement, in comparison with conventional methods, may better predict the risk of blood loss and the need for transfusion products. Improved prediction could contribute to safer anticoagulation, since CAT quantifies the (anti) coagulant effects during CPB, to more optimal haemostasis, to a decrease in excessive bleeding and to improved transfusion management.

## Methods

### Study population

The study was approved by the local medical ethics committee (METC aZM/UM), and written informed consent was obtained. In total, 30 male patients undergoing elective first-time coronary artery bypass grafting (CABG) were enrolled. Exclusion criteria were age < 18 years, use of preoperative anticoagulation (excepting aspirin) within the preceding 5 days, known coagulopathy, impaired renal function, liver diseases resulting in elevated liver function tests and redo surgery.

### Anticoagulation and CPB

An initial dose of 300 IU/kg of body weight of heparin (Heparin Leo, Leo Pharmaceutical Products BV, Weesp, the Netherlands) was injected into a central venous line before initiation of CPB. The kaolin activated clotting time (ACT) was measured and, if the value was ≥400 s, CPB was initiated. If necessary, additional heparin was added. At the end of CPB, heparin was reversed by protamine chloride (Valeant Pharmaceuticals, Eschborn, Germany) at a 1:1 ratio of the loading dose.

All components of the CPB system were poly-2-methoxyethylacrylate coated (Terumo).

The priming of the CPB circuit included 1,300 ml of 4% Gelofusine, 200 ml 20% mannitol, 100 ml 20% human albumin, 50 ml 8.4% NaHCO_3_. and 6,500 IU heparin Leo. Retrograde autologous priming was used in most of the cases to reduce the priming volume by 200–500 ml, resulting in less haemodilution. Normothermic perfusion was used during CPB. Pericardial, pleural and residual blood of the CPB circuit after termination of CPB was drained and washed with a cell saver device. The transfusion trigger during CPB was set at a haematocrit below 23%.

### Blood samples

Blood samples were taken at: T1) pre-bypass before heparin administration; and, T2) 5 min after protamine administration. T1 measurements will give information about the predictive value of the parameters prior to haemostatic interventions related to CPB which could suggest differences in baseline haemostatic capacity of the blood between the patients, whereas T2 measurements may give a prediction of blood loss after CPB including the influence of all haemostatic disturbances related to CPB. Blood samples were withdrawn from the arterial line after discarding the first 10 ml.

### Thrombin generation

Arterial blood samples were collected into trisodium citrate and analyzed with CAT as previously reported [9]. TG was measured both in platelet-rich plasma (PRP) and in platelet-poor plasma (PPP) to see the influence of platelets on TG. PRP was used within 1h after blood withdrawal. PPP was stored at −80°C until further analysis. The CAT assay was measured in a prewarmed plate fluorometer (Ascent reader, Thermolabsystems OY, Helsinki, Finland). To each well, 80 μl of plasma was added in combination with the trigger: for PPP, the trigger was 30 p*M* of recombinant tissue factor (rTF) and phospholipid vesicles in Hepes-buffered saline, for PRP the trigger was 20 μl of 3 p*M* of rTF without added phospholipids. Data were analyzed using Thrombinoscope™ software (Thrombinoscope bv, Maastricht, the Netherlands). CAT parameters which are used to determine correlation of TG with postoperative blood loss are: 1) *lag time* (min): the initiation phase of clotting which equals the clotting time; 2) *peak height* (nM): the maximal amount of thrombin formed; 3) *endogenous thrombin potential (ETP)* (nM*min): the area under the curve representing thrombin generation and decay in time; and 4) *time to peak* (min): the time needed to achieve the peak height.

### ACT and laboratory tests

Besides CAT measurements, ACT and laboratory parameters haematocrit, haemoglobin, thrombocytes, fibrinogen, antithrombin, D-dimers, prothrombin time (PT) and activated partial thromboplastin time (aPTT) were determined at both time points to assess patient’s haemostatic profile.

### Postoperative blood loss

Blood loss was measured at fixed time points postoperatively determined by chest tube drainage after closing the chest until 20 hours after surgery. The amount of transfusion products (packed red cells, thrombocytes or fresh frozen plasma) was recorded until 20 hours after surgery.

### Statistical analysis

Data were defined as continuous or categorical variables. Categorical variables are expressed as percentages and continuous variables as mean ± standard deviation (SD). Patients were divided into two groups with high versus low blood loss, where the median value of blood loss volume at 20 hours postoperatively was used as cut-off value. Both groups were compared with respect to patient characteristics, medication used pre- and intraoperatively, infusion solutions, mean time on bypass, amount of transfusion products, mean values of CAT parameters and other laboratory parameters. The Chi-square test was used for comparison of proportions and the Student’s t-test for independent samples for comparison of mean values. The ability of CAT and laboratory parameters to discriminate between the two groups was also evaluated by the construction of a receiver operating characteristic (ROC) curve. The corresponding area under the curve (AUC) with 95% confidence interval was used to quantify the predictive value of the parameters. Multivariate linear regression analysis was performed to evaluate the independent effects of parameters. Statistical analysis was performed with SPSS for Windows 16.0 (SPSS, Inc., Chicago, IL, USA). P-values ≤ 0.05 were considered to indicate statistical significance.

## Results

Thirty male patients were enrolled into the study. One patient was excluded because an exceptionally long term adhesiolysis was necessary to achieve access to the heart resulting in excess blood loss. The distribution of age, preoperative use of aspirin, and intraoperative data relating to the 29 patients are shown in Table 1. In addition to presenting data of the total group, we divided patients in two groups using as cut-off value the median volume of postoperative blood loss until 20 hours (group 1: patients with median blood loss <930 ml; group 2: patients with median blood loss ≥930 ml). The actual blood loss in group 1 was 620 ml, and 1205 ml in group 2. The sample size was based on feasibility considerations. Based on this sample size and the observed standard deviation in group 1 (sd= −/+ 160 ml) the power to detect a clinically relevant increase in mean blood loss by at least 50% (from 620 ml to 930 ml) was higher than 90%.

**Table 1:** Distribution of patient characteristics and perioperative variables according to postoperative blood loss

	**All patients**	**Group 1**	**Group 2**	**P-value**
	**N=29**	**N=14**	**N=15**	
Age (years)	65 ± 10	67 ± 9	64 ± 11	0.423
Aspirin exp. preop. *n* (%)	27 (93,1%)	14 (100%)	13 (86,7%)	0.157
*Intraoperatively*				
Tranexam. acid *n* (%)	14 (48,3%)	9 (64,3%)	5 (33,3%)	0.096
Heparin*-total* (IU/kg)	336 ± 54	361 ± 66	314 ± 26	0.023
Protamine-*total* (mg/kg)	3.35 ± 0.52	3.55 ± 0.61	3.17 ± 0.37	0.048
Crystalloids (ml/kg)	16.9 ± 6.5	19.8 ± 6.4	14.2 ± 5.6	0.018
Colloids (ml/kg)	18.6 ± 5.2	17.2 ± 4.8	19.9 ± 5.4	0.167
PRC (units)*	0.0 (0.0-1.0)	0.0 (0.0-1.0)	0.0 (0.0-1.0)	0.591
FFP (units)	0 ± 0	0 ± 0	0 ± 0	1
PLT (units)*	0.0 (0.0-1.0)	0.0 (0.0-1.0)	0.0 (0.0-0.0)	0.301
PRC cell saver (ml/kg)	5.7 ± 2.0	5.0 ± 1.3	6.4 ± 2.3	0.048
Time on bypass (min)	76 ± 20	70 ± 18	81± 22	0.160
Time X-clamp (min)	48 ± 16	45 ± 14	52 ± 18	0.259
*Postoperatively*				
Blood loss 20 h (ml)	922 ± 384	620 ± 160	1205 ± 307	<0.001
PRC (units)*	1.00 (0.0-5.0)	0.0 (0.0-2.0)	2.0 (0.0-2.0)	0.002
FFP (units)*	0.0 (0.0-2.0)	0.0 (0.0-0.0)	0.0 (0.0-2.0)	0.164
PLT (units)*	0.0 (0.0-1.0)	0.0 (0.0-0.0)	0.0 (0.0-1.0)	0.020
Rethoracotomy *n* (%)	1 (3,4%)	0 (0%)	1 (6,7%)	0.326

Data shown are mean ± SD. Comparison between the groups was done with student t-test; * median and range are presented instead of mean and SD; colloids = infusion volume and priming volume; *FFP* fresh frozen plasma, *PLT* platelets, *PRC* packed red cells.

Differences in both medication and infusion (Table 1) could result in changes in the haemostatic capacity of the blood leading to differences in thrombin generation and, as a consequence, in blood loss. There was no significant difference between aspirin exposure preoperatively between the two groups. Patients in group 2 were infused less crystalloids, and more autologous packed red cells derived from intraoperative cell salvage. Moreover, this group was administered less heparin (total amount of both pre and during CPB) and as a consequence less protamine. Time of bypass and aorta cross clamping were increased in group 2, but differences were not statistically significant.

### Thrombin generation parameters measured in platelet-rich and platelet-poor plasma

**Figure 1:**
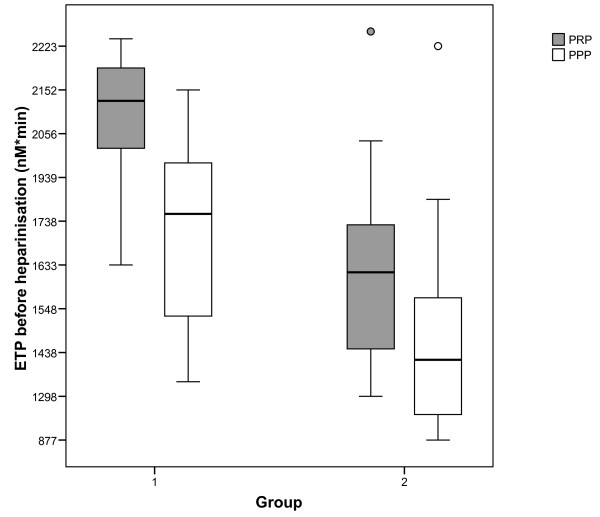
**ETP on T1 in PRP and PPP in both groups.** Boxplot showing median, interquartile range and range of ETP measured before heparinisation in patients of group 1 and 2 on x-axis. P-value of comparison of ETP in PRP between the groups is <0.001, p-value of comparison of ETP in PPP between the groups is 0.004; ETP=endogenous thrombin potential; PPP=platelet poor plasma; PRP=platelet rich plasma.

**Figure 2:**
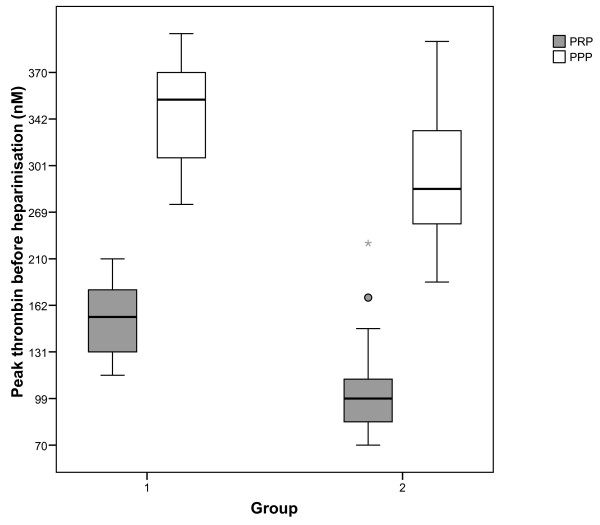
**Peak thrombin on T1 in PRP and PPP in both groups.** Boxplot showing median, interquartile range and range of peak thrombin measured before heparinisation in patients of group 1 and 2 on x-axis. P-value of comparison of peak thrombin in PRP between the groups is 0.004, p-value of comparison of peak thrombin in PPP between the groups is 0.014; PPP=platelet poor plasma; PRP=platelet rich plasma.

The mean values (± SD) of TG parameters on T1 and T2 in both PRP and PPP in group 1 versus group 2 are presented in Table 2. Patients of group 2 had a significantly lower ETP and peak thrombin both on T1 and T2. Between group differences in mean values for lag time and time to peak were smaller and not statistically significant. The distributions of ETP and peak values measured in PRP and PPP within groups are shown in Figures 1 and 2.

**Table 2:** TG parameters on T1 and T2, and their association with postoperative blood los

	**T1 (N=29)**	**T2 (N=29)**	**T1**			**T2**			**ROC analysis**	
			**Group 1 (N=14)**	**Group 2 (N=15)**	**p**	**Group 1 (N=14)**	**Group 2 (N=15)**	**p**	**T1 AUC**	**T2 AUC**
*PRP:*										
Lag time (min)	8.7 ± 3.0	8.7 ± 3.2	7.9 ± 1.4	9.4 ± 3.8	0.186	9.0 ± 3.1	8.5 ± 3.4	0.700	0.452 (0.232-0.672)	0.543 (0.322-0.764)
ETP (nM* min)	1881 ± 359	1524 ± 264	2104 ± 207	1672 ± 348	0.000	1649 ± 197	1407 ± 270	0.011	0.881 (0.741-1.021)	0.800 (0.630-0.970)
Peak (nM)	133 ± 42	73 ± 19	155 ± 30	112 ± 42	0.004	82 ± 18	64 ± 17	0.010	0.855 (0.697-1.013)	0.776 (0.605-0.947)
Time to peak (min)	19.3 ± 4.8	20.3 ± 5.6	18.4 ± 3.5	20.2 ± 5.7	0.315	20.1 ± 5.4	20.5 ± 6.0	0.870	0.381 (0.170-0.592)	0.493 (0.273-0.713)
*PPP:*										
Lag time (min)	2.5 ± 0.5	4.2 ± 0.9	2.4 ± 0.4	2.6 ± 0.6	0.368	4.2 ± 1.0	4.1 ± 0.9	0.926	0.450 (0.236-0.664)	0.502 (0.287-0.718)
ETP (nM*min)	1587 ± 355	1508 ± 297	1774 ± 279	1412 ± 336	0.004	1610 ± 246	1411 ± 315	0.070	0.805 (0.639-0.971)	0.690 (0.493-0.888)
Peak (nM)	321 ± 63	241 ± 36	350 ± 48	295 ± 64	0.014	254 ± 31	230 ± 36	0.070	0.771 (0.597-0.946)	0.700 (0.312-0.745)
Time to peak (min)	4.7 ± 0.7	6.7 ± 1.1	4.6 ± 0.4	4.8 ± 0.9	0.532	6.8 ± 1.3	6.6 ± 1.0	0.588	0.502 (0.282-0.723)	0.529 (0.312-0.745)

Data shown are mean ± SD. Comparison between the groups was done with student t-test. *T1* before heparinisation, *T2* after protamine administration, *AUC* area under the curve, *ETP* endogenous thrombin potential, *PPP* platelet poor plasma, *PRP* platelet rich plasma, *ROC* receiver operating curve.

The AUCs as measures for ability to discriminate between the groups were highest for ETP and peak in PRP at T1 with AUC (95% CI) of 0.881 (0.741-1.021) and 0.855 (0.697-1,013) respectively.

### Laboratory tests

The mean values of haemoglobin, haematocrit, platelet number, fibrinogen, d-dimers, antithrombin, PT, aPTT, and ACT at T1 and T2 are shown in Table 3. A significant difference between the groups was only found for platelet number (p=0.002) with lower platelet count in group 2. Differences were also seen for fibrinogen and D-dimers with lower mean values in the group with blood loss ≥930 ml, but only the difference in fibrinogen at T2 achieved statistical significance (p=0.045). Furthermore, it can also be observed that patients with high blood loss had significantly lower haematocrit (%) at T2.

**Table 3:** Laboratory parameters on T1 and T2, and their association with postoperative blood loss

	**T1 (N=29)**	**T2 (N=29)**	**T1**			**T2**			**ROC analysis**	
			**Group 1 (N=14)**	**Group 2 (N=15)**	**p**	**Group 1 (N=14)**	**Group 2 (N=15)**	**p**	**T1 AUC (95% CI)**	**T2 AUC (95% CI)**
Hemoglobin (mmol/l)	7.8 ± 0.6	5.4 ± 0.5	7.7 ± 0.5	7.8 ± 0.8	0.435	5.6 ± 0.5	5.3 ± 0.4	0.098	0.377 (0.165-0.590)	0.714 (0.517-0.912)
Hematocrit (%)	37.2 ± 3.3	25.9 ± 2.3	36.8 ± 2.6	37.7 ± 3.8	0.475	26.8 ± 2.2	25.1 ± 2.2	0.044	0.401 (0.186-0.617)	0.732 (0.540-0.924)
Platelet number (*10^9^/l)	215 ± 61	128 ± 42	249 ± 57	184 ± 48	0.002	152 ± 40	105 ± 31	0.002	0.834 (0.690-0.979)	0.857 (0.705-1.009)
Fibrinogen (g/l)	2.9 ± 0.7	1.7 ± 0.4	3.1 ± 0.8	2.7 ± 0.5	0.198	1.9 ± 0.5	1.5 ± 0.3	0.045	0.627 (0.419-0.835)	0.703 (0.512-0.894)
D-dimers (ng/ml)	700 ± 782	1109 ± 1146	949 ± 1063	467 ± 235	0.119	1083 ± 1069	1132 ± 1251	0.909	0.654 (0.442-0.866)	0.547 (0.333-0.761)
Antithrombin (%)	80.9 ± 11.5	49.6 ± 6.6	80.1 ± 7.8	81.6 ± 14.4	0.727	50.1 ± 7.1	49.0 ± 6.3	0.651	0.440 (0.229-0.651)	0.574 (0.364-0.783)
PT (sec)	11.5 ± 0.5	15.4 ± 1.5	11.4 ± 0.6	11.5 ± 0.4	0.417	15.2 ± 1.3	15.7 ± 1.6	0.341	0.425 (0.205-0.646)	0.373 (0.168-0.577)
aPTT (sec)	28.9 ± 3.2	34.6 ± 5.5	28.9 ± 3.9	28.8 ± 2.7	0.922	34.1 ± 5.6	35.1 ± 5.5	0.634	0.498 (0.276-0.719)	0.406 (0.197-0.615)
ACT (sec)	132 ± 15	140 ± 17	134 ± 18	131 ± 12	0.625	140 ± 15	141 ± 19	0.875	0.498 (0.281-0.714)	0.487 (0.275-0.698)

Data shown are mean ± SD. Comparison between the groups was done with student t-test. *T1* before heparinisation, *T2* after protamine administration, *ACT* activated clotting time, *aPTT* activated partial thromboplastin time, *PT* prothrombin time.

The highest AUC values were observed for platelet number with an AUC (95% CI) of 0.834 (0.690-0.979) at T1 and an AUC (95% CI) of 0.857 (0.705-1.009) at T2. It can also be observed that the AUC of haemoglobin and haematocrit is higher at T2 than at T1 with AUC (95% CI) of 0.714 (0.517-0.912); and 0.732 (0.540-0.924), respectively. AUCs for these parameters at T1 are low. The AUCs associated with fibrinogen and d-dimers are not impressive at both time points with AUCs ranging from 0.547 to 0.703, and the AUC of the variables, aPTT, PT and ACT are below 0.500.

### Independent effects of platelet count and CAT parameters

A multivariate linear regression model with both platelet count and ETP/peak as independent variables and blood loss as dependent variable was performed to evaluate the independent effects of TG parameters after adjustment for differences in platelet count between group 1 and 2. These analyses indicate that higher values of CAT parameters remain associated with lower blood loss. Results for measurements in PRP on T1 are shown in Tables 4 and 5, and results for the other measurements are similar.

**Table 4:** Independent effects of platelets and ETP in PRP on blood loss by linear regression analysis

	**Univariate**			**Multivariate**		
	**B Unstandardized**	**B Standardized**	**p-value**	**B Unstandardized**	**B Standardized**	**p-value**
ETP (nM* min)	−0.429	−0.401	0.031	−0.322	−0.301	0.144
Platelet number (*10^9^/l)	−2.223	−0.354	0.060	−1.325	−0.211	0.301

*B* regression coefficient, *ETP* endogenous thrombin potential.

**Table 5:** Independent effects of platelets and peak-thrombin in PRP on blood loss by linear regression analysis

	**Univariate**			**Multivariate**		
	**B Unstandardized**	**B Standardized**	**p-value**	**B Unstandardized**	**B Standardized**	**p-value**
Peak (nM)	−0.2897	−0.319	0.092	−2.026	−0.223	0.253
Platelet number (*10^9^/l)	−2.223	−0.354	0.060	−1.737	−0.276	0.159

*B* regression coefficient.

## Discussion

The present data suggest that the CAT variables ETP and peak thrombin correlate well with the clinically observed bleeding tendency postoperatively in cardiac surgical patients. Patients of group 2 had a significantly lower ETP and peak thrombin, both pre (PRP and PPP) and post CPB (only PRP), than patients in group 1. Concerning the other laboratory parameters, platelet number was the only individual variable measured *pre*-CPB predicting blood loss postoperatively. Post CPB, besides low fibrinogen a low platelet number was also predictive for blood loss.

Recently Coakley and co-workers demonstrated that TG both pre- and postoperatively could potentially be used to identify patients at an increased risk of bleeding post-CPB [2]. However, they only measured TG in PPP, excluding the influence of platelets, which play a very important role in maintaining normal haemostatic function. It is more representative of the in vivo situation to include the procoagulant functions of platelets [10].

Conventional clot-based methods, have the disadvantage of measuring only the moment of clot formation and ensuing changes in the properties of the clot. Clot formation is only one of the many functions of thrombin and not necessarily the most important one. Thrombin also activates platelets, and furthermore, besides procoagulant effects it has also anticoagulant effects following its binding to thrombomodulin. Ex vivo TG tests, like CAT, measure the haemostatic function of the blood, determined by simultaneous prothrombin activation and thrombin inactivation [9]: it measures the remaining capacity of blood to generate a thrombin burst indicating an increased risk of thrombosis or bleeding [11]. In contrast, *in vivo* TG, revealed by products like prothrombin fragment F1+2, thrombin antithrombin complex, and d-dimers, provides indications of TG that has already occurred [12] at the moment of blood collection.

Since the lag time represents the same aspect as in standard coagulation assays, this is comparable with aPTT and PT. Our results showed that both aPTT and PT as well as lag time were not different between the groups. These tests terminate with endpoints that occur with less than 5% of the reaction complete [13]. Apparently, the present data strengthen the notion that it is not the initiation phase of clotting but the propagation phase that determines haemostasis and blood loss after cardiac surgery.

Time to peak, also a time dependent variable, also did not show any significance in predicting blood loss. Peak thrombin and ETP, reflecting the amount of thrombin generated, give a more accurate evaluation of coagulability.

In group 2, patients had a lower mean platelet number both before heparinisation and after protamine administration. Platelets are important in the blood coagulation process and play an important role in TG by providing a procoagulant membrane surface, and hence supporting the formation of more thrombin [14]. We observed in this study that reduced platelet count was also predictive of blood loss although the latter outcome occurs within the normal ranges of platelet count. It is worth noting however that platelet count cannot detect abnormality in the coagulation system. CAT, in contrast, is a global functional test that reflects the coagulation profile of the patient in the absence and presence of platelets. Bleeding tendency is related to the hemostatic conditions in pro- and anticoagulant factors, platelets, vessel wall and the fibrinolytic system. TG in PRP reflects the major part of this physiological clotting system, including the interaction between platelets and the clotting system.

Concerning coagulation factors we only measured fibrinogen. This factor demonstrated to be higher both pre- and postoperatively in group 1, but the difference was only statistically significant postoperatively. In the study of Coakley [2] coagulation factor levels were determined postoperatively in groups that either bled more than 1L or less than 1L. Fibrinogen level was the same in both groups, in contrast with our results. Karlsson et al. [15] investigated the relationship between preoperative fibrinogen plasma concentration and postoperative bleeding after CABG surgery. Their main finding was that higher preoperative fibrinogen was correlated with less blood loss postoperatively. Despite of the important contribution of fibrinogen in the coagulation process, our study results indicate that fibrinogen level is less strongly associated with blood loss than TG parameters. This is remarkable because, although fibrinogen is depleted before 5% of all thrombin is formed [11], thrombin bound to fibrin has a positive feedback activation on the coagulation system and is protected from inactivation to the action of antithrombin and heparin [16].

Remarkable is the significantly higher dose of intraoperative heparin in group 1. Most probably a higher dose of heparin is administered in this group as a result of a relatively high platelet count in this group [17]: the concentration of heparin required to inhibit or delay coagulation is directly related to the number of platelets [18], probably due to the capacity of platelet released platelet factor 4 neutralizing heparin [19,20].

Patients who bled more, were administered proportionally less crystalloids and more colloids. Colloids are known to affect clot formation by reducing fibrinogen concentration and disturbing fibrin polymerization [21] demonstrated by thromboelastography assay. Schols et al. found that colloids reduce the formation of fibrin clots but affect TG only at clinically irrelevant high concentrations [22]. The volume of colloids administered was not significantly different between the groups.

Group 2 is characterized by a higher supplementation of transfusion products. In addition, these patients had a higher intraoperative blood loss, as shown by a higher amount of PRC volume processed by cell salvage. Despotis et al. also demonstrated that greater volumes of intraoperative salvaged red cells were associated with excessive blood loss and use of blood products [23], explained by the fact that greater volumes of salvaged red cells primarily reflects excessive blood loss intraoperatively but proceeding postoperatively. Another explanation may be that extensive intraoperative cell salvage caused a significant loss of platelets and plasma resulting in postoperative bleeding.

The strength of this study is that CAT, performed preoperatively, is able to predict blood loss after cardiac surgery. Preoperative measurements (T1) exclude the haemostatic effects caused by exposure of the blood to different non-physiological conditions. The predictive value of CAT performed postoperatively (T2) for blood loss will be influenced by a lot of interventions like heparinisation, fluid administration (crystalloid and/or colloid), administration of tranexamic acid and protamine, time on bypass, and transfusion product requirements intraoperatively. All these factors interfere at different levels in the haemostatic balance of the patients what makes the prediction of blood loss by CAT parameters weaker at T2 (AUCs are lower at T2).

The main limitation of the study is the low number of participants (n=30). P-values in the multivariate linear regression models were higher or even no longer statistically significant, possibly due to small sample size. The results should be considered as preliminary. Changes in haemostatic factors would have been more reliable in a larger number of patients. Another limitation of the study is that it could not account for variations in routine practice in the ICU that may have influenced blood loss, such as amount and timing of platelet and plasma transfusions. Especially in the bleeding group the amount of blood loss should be influenced by platelet and plasma transfusion. Therefore, additional larger clinical, and preferable intervention, studies are required to establish profound clinical relevance.

## Conclusions

The key finding is that the Calibrated Automated Thrombography assay, performed preoperatively, provides information predictive for non-surgical blood loss after cardiac surgery, and might be considered as a standard screening test to assess the haemostatic condition preoperatively. Improved haemostatic management of patients undergoing cardiac surgery, especially high risk patients, requires more knowledge on the changes in TG, and on proactive interventions like administration of haemostatic-augmenting pharmacologic agents or blood components. Identifying patients at increased risk of bleeding will become easier because techniques for measuring TG in whole blood as a bedside method are in development, so that the outcome of a study with the CAT can, in the future, be more easily applied in clinical settings.

## Abbreviations

CAT: Calibrated automated thrombography; TG: Thrombin generation; PRP: Platelet rich plasma; PPP: Platelet poor plasma; ETP: Endogenous thrombin potential; CPB: Cardiopulmonary bypass; TEG: Thromboelastography; CAB: Coronary artery bypass grafting; ACT: Activated clotting time; rTF: Recombinant tissue factor; PT: Prothrombin time; aPT: Activated partial thromboplastin time; ROC: Receiver operating curve; AUC: Area under the curve; PRC: Packed red cells; FFP: Fresh frozen plasma; PLT: Platelets.

## Competing interests

The authors declare that they have no competing interests.

## Authors’ contributions

YB, RA, HC, CH, and BM designed the study. YB collected the clinical data. RA analysed and interpreted the data. SB carried out the CAT assays. PN performed the statistical analysis. YB drafted the manuscript. RA, HC, CH, PW, JM and BM made critical revision of the manuscript for important intellectual content. All authors read and approved the final manuscript.
